# A New Subgenus and Species of *Priochirus* from Mid-Cretaceous Kachin Amber (Coleoptera: Staphylinidae: Osoriinae) †

**DOI:** 10.3390/insects13060513

**Published:** 2022-05-30

**Authors:** Yuan Peng, Rixin Jiang, Chao Shi, Xiaoxuan Long, Michael S. Engel, Shuo Wang

**Affiliations:** 1 College of Marine Science and Biological Engineering, Qingdao University of Science and Technology, Qingdao 266042, China; yuanpeng1027@163.com (Y.P.); chaoshi@qust.edu.cn (C.S.); budinger0620@163.com (X.L.); 2 The Provincial Key Laboratory for Agricultural Pest Management of Mountainous Region, Institute of Entomology, Guizhou University, Guiyang 550025, China; maoshuwuyouzhi@163.com; 3 Natural History Museum and Department of Ecology and Evolutionary Biology, University of Kansas, Lawrence, KS 66045, USA; msengel@ku.edu

**Keywords:** Staphylinoidea, new species, Burmese amber, fossil insects, Myanmar

## Abstract

**Simple Summary:**

Despite the extant diversity of the rove beetle subfamily Osoriinae, its fossil record remains meagre. The present study reports a new species *Priochirus (Eopriochirus) trisclerite* subgen. et sp. nov. from Kachin amber (mid-Cretaeous: Cenomanian, ca. 99 Ma). The discovery not only enriches the fossil record of Osoriinae but adds to our understanding of its ancient origin and diversification. With new species described in the present paper, 3 species of osoriines are known from Mesozoic Kachin amber.

**Abstract:**

As one of the largest families of beetles (Coleoptera), the Staphylinidae (rove beetles and their relatives) are rich not only in extant species but also in a comparatively robust fossil record. Despite this preponderance of available fossil material, fossils of the diverse subfamily Osoriinae remain rare. Here, we describe a new ososriine species, *Priochirus trisclerite* sp. nov., from the mid-Cretaceous amber of Myanmar. The new specimen is similar to the only other definitive fossil of the genus, *Priochirus thayerae* Yamamoto 2019, and both are placed in the extinct subgenus *Eopriochirus* subgen. nov. The new species differs noticeably in a number of morphological details in relation to the submentum, gular sutures and protibial crenulae. The new fossil provides further evidence for understanding the radiation of staphylinoid beetles.

## 1. Introduction

The subfamily Osoriinae, also known as “unmargined rove beetles”, is one of the most varied subfamilies and currently includes more than 2390 species in 118 genera [1,2,3,4]. They are widely distributed in all biogeographic regions except Antarctica, but they are distinctly more diverse in tropical areas [1,2]. Modern osoriines are considered mycophagous or saprophagous and are usually found in decaying wood or under the bark of decomposing trees although little is known about the details of their behavior or bionomics [2,3,5]. Osoriines can be distinguished from their relatives most easily by the unmargined abdomen; i.e., each abdominal segment lacks paratergites owing to the complete fusion of the tergum and sternum to form a continuous ring [5]. Although a few other groups of staphylinids also may have unmargined abdomens, such as certain genera of Paederinae and Euaesthetinae and some species of *Stenus* Latreille, they can be distinguished from Osoriinae by the latter’s slender, falcate mandibles and concealed antennal insertions [5].

In recent years, the fossil record of Osoriinae has increased gradually, ranging in age from the Miocene to mid-Cretaceous. Zhang [6] described a Miocene fossil species, *Sinolispinodes torosus* Zhang, from Shandong Province, China, but placed it originally among the Oxytelinae. It was later recognized as an osoriine owing to the lack of paratergites and was therefore transferred to the subfamily [1,7]. Additionally, from the Miocene, Irmler [8] described several fossil species from Dominican amber, including the extinct genus *Lispinomimus* Irmler, two named species (*Thoracophorus palaeobrevicristatus* Irmler and *Nacaeus dominicanensis* Irmler), and five unnamed species of the genera *Liberiana* Blackwelder, *Osoriellus* Fagel, and *Neosorius* Fagel. Subsequently, Ortega-Blanco et al. [9] described a new genus and species, *Paleosorius cambayensis* Ortega-Blanco, Chatzimanolis, and Engel, from the Eocene Camby amber of India. From the Mesozoic, only three osoriines have been discovered, all from mid-Cretaceous Kachin amber [10,11]. A further fossil, *P. comes* Greenslade, is excluded here as it was only briefly mentioned by Greenslade [12], but the diagnostic information is insufficient as to consider it anything other than *Incertae sedis*.

Herein, we describe a fourth Cretaceous fossil species of osoriine, *Priochirus tressclerite* sp. nov., from Kachin amber. The finding of discovery of further osoriine fossils provides an added glimpse into the evolutionary history of the subfamily, a group that had apparently diversified significantly by the Late Cretaceous.

## 2. Materials and Methods

The amber specimen studied herein was found in the Hukawng Valley, Kachin State, northern Myanmar (26°21′33.41″ N, 96°43′11.88″ E) [13]. The age of Kachin amber has been dated to 98.79 ± 0.62 Ma based on U-Pb zircons (earliest Cenomanian) [14]. We are mindful of the ethical concerns pertaining to Kachin amber and we declare that the specimen reported herein was collected prior to 2015, and it is, therefore, free of current ethical concerns surrounding the post-June 2017 acquisition of amber from the region [15,16,17]. The amber piece is conserved in the research collections of the Qingdao University of Science and Technology, Qingdao, China (QUST).

For a better view, a handheld engraving tool was used to cut the amber piece and sandpapers of varying grain sizes and rare earth polishing powder were used to polish the piece. Observations were made using a high-resolution stereomicroscope (D-07747 Jena, Leica, Germany), while photographs were taken using a Canon 5D SR camera with an MP-E 65mm f/2.8 1–5× macro lens, while a Canon MT-26EX twin flash was used as the light source. Zerene Stacker v. 1.04 was used to produce extended depth images. All of the final images were cleaned and arranged in Adobe Photoshop CS5 Extended (Adobe Systems, San Jose, CA, USA).

## 3. Systematic Palaeontology

Family Staphylinidae Latreille, 1802

Subfamily Osoriinae Erichson, 1839

Tribe Leptochirini Sharp, 1887

Genus *Priochirus* Sharp, 1887

Subgenus *Eopriochirus* Peng, Jiang, Engel and Wang, subgen. nov.

**Figure 1:**
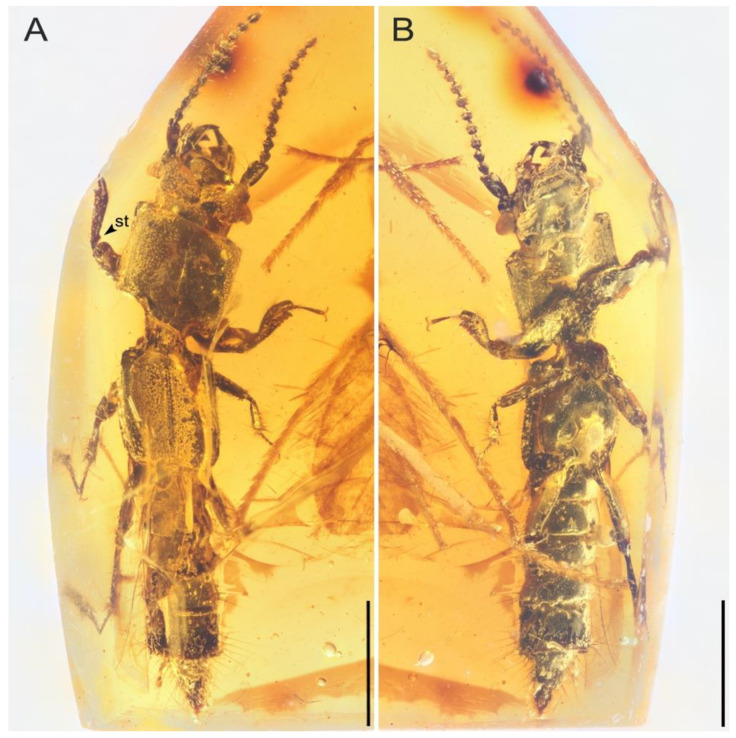
*Priochirus trisclerite* sp. nov., holotype, QUST-SHUO-0003. (**A**) Dorsal habitus. (**B**) Ventral habitus. Abbreviations: st, subtriangular teeth. Scale bars = 1 mm.

**Figure 2:**
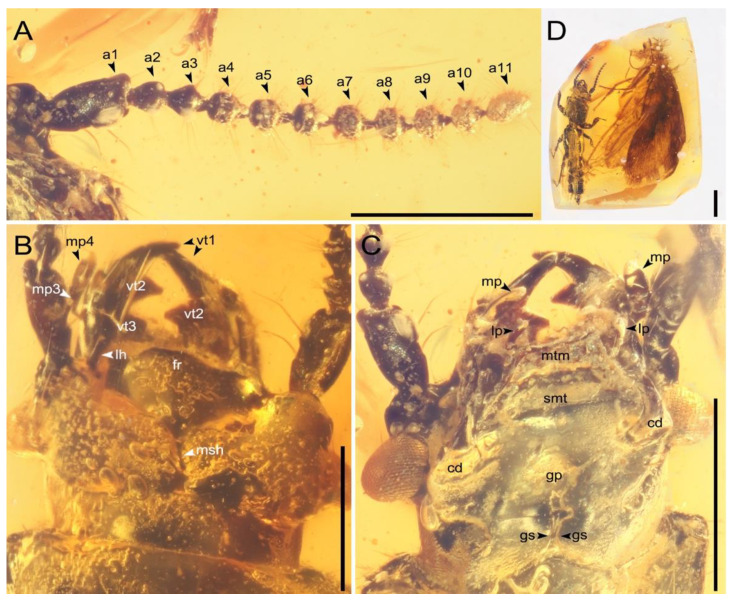
Entire amber piece with holotype and detailed of head of *Priochirus trisclerite* sp. nov., holotype, QUST-SHUO-0003. (**A**) Right antenna, ventral view. (**B**) Head, dorsal view. (**C**) Head, ventral view. (**D**) Piece of amber with holotype (trichopteran syninclusion at right). Abbreviations: a1–11, antennomeres; cd, cardo of maxilla; fr, frons; gp, gular plate; gs, gular suture; lh, lateral horn of head; lp, labial palpus; mp, maxillary palpus; mp3–4, maxillary palpomeres; msh, median longitudinal sulcus; mtm, mentum; smt, submentum; vt1–3, ventral teeth of mandible. Scale bars for (**A**–**C**) = 0.5 mm; (**D**) = 1 mm.

**Figure 3:**
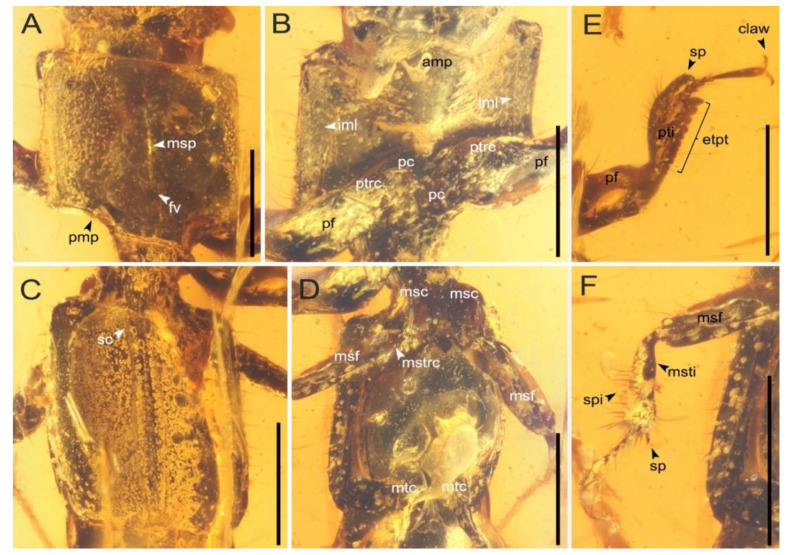
Thorax and legs of *Priochirus trisclerite* sp. nov., holotype, QUST-SHUO-0003. (**A**) Pronotum, dorsal view. (**B**) Pronotum in ventral view. (**C**) Mesoscutellum and elytra, dorsal view. (**D**) Meso- and metathorax, ventral view. (**E**) Right foreleg, dorsal view. (**F**) Right midleg, ventral view. Abbreviations: amp, anterior margin of prosternum; etpt, external teeth along protibia; fv, fovea; iml, inferior marginal line of pronotal hypomeron; msc, mesocoxa; msf, mesofemur; msp, median longitudinal sulcus; msti, mesotibia; mtc, metacoxa; mstrc, mesotrochanter; pc, procoxa; pf, profemur; pmp, posterior marginal process; pti, protibia; ptrc, protrochanter; sc, mesoscutellum; sp, spur; spi, spines. Scale bars = 0.5 mm.

**Figure 4:**
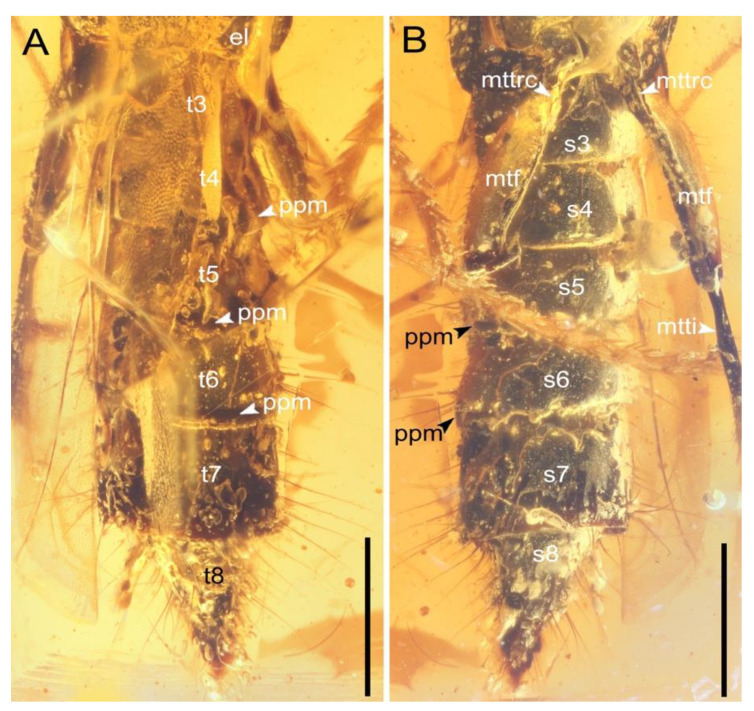
Abdomen of *Priochirus trisclerite* sp. nov., holotype, QUST-SHUO-0003. (**A**) Abdomen, dorsal view. (**B**) Abdomen, ventral view. Abbreviations: el, elytron; mtf, metafemur; mtti, metatibia; mttrc, metatrochanter; ppm, punctation on posterior margin; s3–8, sternites III–VIII; t3–8, tergites III–VIII. Scale bars = 0.5 mm.

Type species: *Priochirus (Eopriochirus) trisclerite* sp. nov.

(Figure 1, Figure 2, Figure 3 and Figure 4)

urn:lsid:zoobank.org:pub:482D2E5F-75AE-43A0-90A7-05BE3188C39C

**Diagnosis.** The new subgenus can be distinguished by the following combination of characteristics: head with a single pair of lateral horns, horns short and blunt (not long, nor with acute or bifid apices); broad depression on head lacking; mandibles relatively symmetrical.

**Etymology.** The new subgeneric name is a combination of Ancient Greek *Ēṓs* (*Ἠώς*, the mythological goddess of dawn, and an allusion to early or ancient) and the generic name *Priochirus* Sharp. The gender of the name is masculine.

**Included species.** Currently, the subgenus includes the type species and *Priochirus thayerae* Yamamoto, 2019.

***Priochirus (Eopriochirus) trisclerite* sp. nov.**

**Etymology.** The specific epithet is a combination of the Latin numeric prefix tri– (meaning, “three”) and the noun sclerite (meaning, “hardened part”, and Latinized from Ancient Greek *sklērós/σκληρός*, meaning, “hard” and *–ī́tēs/–ῑ́της*, a suffix indicating *a member of or one connected to*, such that a sclerite is a one of a series of hardened parts), and is a reference to the preservational artifact formed by three small sclerites in segment VI dorsolaterally.

**Material.** Holotype, preserved in an irregularly polygonous piece of amber, labeled, ‘QUST-SHUO-0003’ (QUST); specimen housed in the research collections of the Qingdao University of Science and Technology, Qingdao, China.

**Diagnosis.** Head with one pair of cephalic lateral horns, horn short, blunt (not acute, long, nor bifid apically). Vertex with mediolongitudinal sulcus. Frontal medial margin weakly produced anteriorly and nearly straight (vs. rounded frontal medial margin in *P. thayerae*); submentum distinct; gular sutures wide at both ends and narrow medially; gular plate slightly concave; pronotum with a row of short, sparse bristles along each lateral margin; pronotal posterior margin with a small process; protibia robust, with five large and moderately separated crenulae, followed by smaller, weakly raised denticles, with a subtriangular tooth proximally on inner surface; protibial surface covered with tufts, dense and gradually shortened setae dorsally, with sparse, long setae ventrally and a row of short setae between crenulae and denticles; abdominal segments IV–VI with apical margins punctate dorsally and ventrally, these margins thickened and slightly elevated relative to the remainder of the surface.

**Description.** Body (Figure 1) length 4.7 mm including mandibles; body elongate, subparallel-sided, moderately flattened; surface smooth, glossy, partially covered with long, stout setae; colour dark black (where evident).

Head (Figure 2) relatively small, 0.53 mm long and 0.93 mm wide (including compound eyes), widest across compound eyes; anterior margin between cephalic lateral horns (a.k.a., “teeth”) slightly emarginate, moderately elevated mediolongitudinally, medial margin flattened; vertex with longitudinal sulcus medially, sulcus parallel to lateral margins; neck present, constricted behind compound eyes dorsally and laterally. Compound eyes positioned laterally, bulging (exophthalmic), rather large, strongly projecting laterally, slightly produced posteriorly (likely postmortem deformation). Antenna (Figure 2A) long, moniliform, close to compound eyes, with 11 antennomeres, antennal insertions exposed anterior to eyes; antennomere I broad and elongated, more robust than remaining antennomeres, slightly dilated apically, 1.7× as long as wide, 2.7× as long and 1.6× as wide as antennomere II; antennomere II irregular fusiform, 1.2× as long as wide, 1.1× as long as and subequal in width to antennomere III; antennomere III fusiform, 1.4× as long as wide, widest near midlength; antennomeres IV–X small, nearly spherical, each almost of nearly same size and shape; antennomere XI elongated, slightly dilated, nearly conical, 1.6× as long as wide, widest medially, width narrowest of all antennomeres; antennomeres covered with setae, antennomeres I–II with sparse setae, antennomere I with a row of two obviously long and thick setae on the inner surface, antennomeres III–XI verticillate, with long, stiff setae. Mandibles (Figure 2B,C) comparatively short, produced anteriorly, right mandible with two teeth visible on ventral mesal margin and a single dorsal tooth (a third ventral tooth could be present but is obscured from sight), left mandible with three teeth on ventral mesal margin and a single dorsal tooth. Maxillary palpus (Figure 2B,C) present, short and slender, tetramerous; palpomere I small, not visible from above; palpomere II elongated, slightly shorter than palpomere IV; palpomere III smallest, spherical; palpomere IV nearly cylindrical, with inconspicuous triangular tip apically; all palpomeres without visible setae. Labial palpus (Figure 2B,C) trimerous, only two palpomeres visible ventrally (bubble prevents further observation of details). Mentum (Figure 2C) trapezoidal, slightly roughened, anterior margin slightly concave inwardly to arcuate, apical angles thickened. Gular sutures (Figure 2C) distinctly separated, wide at both ends and narrow medially, sides asymmetrical, anterior of gula parallel, right edge near midlength obviously curved inward to about semicircular; two rather short, stout setae between gena and compound eye; gular plate (Figure 2C) with large, sub-triangular, slight depression medially.

Pronotum (Figure 3A) rectangular, length 0.84, width 0.88mm, widest at the anterior margin and slightly narrower posteriorly; widest part almost equal to head width and distinctly broader than elytra; narrow, median longitudinal sulcus present (Figure 3A), distinctly expanding posteriorly into rounded fovea (Figure 3A); disc slightly convex dorsally; all angles smooth, anterior pronotal angles more rounded, posterior pronotal angles closer to orthogonal; both sides with a row of short, sparse bristles, dorsal surface glabrous; posterior margin with a small, slightly raised process (Figure 3A). Prosternum transverse, with medially projecting anterior margin (Figure 3B). Pronotal hypomeron (Figure 3B) with moderately fine and straight inferior marginal lines. Procoxal cavities (Figure 3B) closed behind and laterally. Prosternal process short and depressed between procoxae. Mesonotum well developed; mesoscutellum (Figure 3C) subtriangular, longer than wide, with the apex pointed. Elytra (Figure 3C) elongated, rectangular, subparallel, 1.2× as long as wide, narrower than pronotum; surface without striae, carinae, or microsetae (air and dense bubbles prevent observation of finer details); anterolateral angles nearly orthogonal, posterolateral angles nearly rounded; elytral lateral margins with shallow groove (Figure 3D); anterior and lateral margins each with a row of sparse, short, stiff setae.

Abdomen (Figure 4) cylindrical, with six sternites, sternites transverse; segment IV with lateral setation differing from other segments; segments IV–VI subequal in length (Figure 4A); segment VII 1.2× as long as segment VI; segment VIII partially retracted into segment VII, with apex strongly narrowed, subtriangular, 1.3× as long as segment VII, covered with dense, long setae; segments V–VII slightly broadened, with dense, long setae laterally, each segment with longer setae posteriorly than anteriorly, length of longest setae on segment longer than those of preceding segment (for segments V–VII); intersegmental membranes present; segments IV–VI with tergite and sternite fused into a continuous ring, apical margin of the ring with punctation and seemingly thicker and more elevated relative to the remainder of surface (Figure. 4) (thin layer of air makes it difficult to further discern these details).

Legs slender, long; pretarsal claws paired. Procoxae (Figure 3B) contiguous, small; protrochanter (Figure 3B) irregularly conical; profemur (Figure 3B) clavate; protibia (Figure 3E) robust, with five large and moderately separated crenulae, followed by smaller, weakly raised denticles, inner surface of protibia with subtriangular tooth proximally (Figure 3E); protibial surface covered with tufts, dense and gradually shorter setae dorsally, with sparse, long setae ventrally and with a row of short setae between crenulae or denticles; protibial apical spur robust, apex curved ventrally; protarsus (Figure 3E) pentamerous, length of protarsus longer than protibia; protarsomere I smaller than protarsomere V; protarsomere II shortest tarsomere, subtrapezoidal; protarsomere III slightly longer than protarsomere II; protarsomere IV subconical; protarsomeres I–IV progressively and gradually narrowed; protarsomere V longest, largest, clavate, longer than combined lengths of protarsomeres I–IV; protarsomeres III–V with sparse, short setae. Midlegs (Figure 3D,F) slightly shorter than forelegs; mesocoxae (Figure 3D) separated and elongate, outwardly expanded; mesotrochanter (Figure 3D) conical and much smaller; mesofemur slender, subparallel; mesotibia (Figure 3F) with numerous small spines and short setae, with a cluster of long setae near apex, with spur smaller than protibial spur; mesotarsus pentamerous, mesotarsomeres I–III of almost equal sizes, mesotarsomere IV conical, mesotarsomere V not well visible; surface of mesotarsus covered with sparse, long setae. Hind legs (Figure 3D and Figure 4B) robust; metacoxae separated (bubbles prevent further examination); metatrochanter (Figure 4B) nearly conical; metafemur (Figure 4B) long and slender, moderately covered with setae on lateral margin; metatibia (Figure 4B) incomplete and metatarsus absent as preserved.

**Remark.** *Priochirus trisclerite* sp. nov. differs from *P. thayerae*. by the following a series of combined characteristics: the postmentum of *P. trisclerite* is distinct, the gular sutures are wide at both ends and narrow medially and the gular plate is slightly concave. Furthermore, the protonal posterior margin of *P. trisclerite* has a small project, but this is absent in *P. thayerae*; the lateral margin has a row of sparse, short setae rather than the dense, long setae of *P. thayerae*. Abdominal segments IV–VI of *P. trisclerite* have the posterior margins thickened and slightly elevated relative to the other part of the integumental surface, this raised portion bearing distinct, dense punctation dorsally and ventrally. *P. trisclerite* has five large and distinctly separated crenulae, followed by smaller, weakly raised denticles on the protibia, while *P. thayerae* has a row of similar-sized and shaped crenulae on the protibia. Lastly, *P. trisclerite* has a subtriangular tooth proximally on the inner surface of the protibia (Figure 1A), and the apex of the protibia has denser setae.

## 4. Discussion

The new fossil can be placed in the osoriine group of subfamilies owing to the following characteristics: (1) unmargined abdominal segments (i.e., without paratergites and with tergum and sternum completely fused into a solid ring); (2) antennae inserted under shelflike corner of frons; (3) mandibles with multiple teeth apically; (4) abdomen with six visible sternites; (5) protrochantin exposed [5]. The subfamily Osoriinae consists of four tribes, the new species can be placed in the tribe Leptochirini on the basis of its general habitus, contiguous procoxae, protibiae with well-developed teeth along much of the inner surface, and exposed protrochantin [5,18]. Further, the fossil can be placed in the genus *Priochirus* and shares many features with *P. thayerae* by the combination of the following characteristics: broad frontal margin, three mesal teeth on mandibles, smooth and glabrous near the median sulcus of the vertex’s posterior margin (rather than setiferous), anterior half of gular sutures closely parallel, median longitudinal sulcus of pronotum evenly narrowed and posterior end expanded as a fovea, and prosternal process depressed between procoxae [11,18]. *Eopriochirus* subgen. nov. can be distinguished from other subgenera of the genus as follows: differs from *Cephalomerus* Bernhauer, *Euleptarthrus* Jakobson, and *Priochirus* s. str. by the presence of only a single pair of lateral horns on the head [18,19,20]; differs from *Paraborolinus* Nakane and Sawada by the absence of a broad frontal depression on the head [21]; differs from *Peucodontus* Bernhauer by with the reduced and blunt lateral horns and rather symmetrical mandibles [12,22,23]. Despite the considerable similarity between the two species of *Eopriochirus*, it is not difficult to distinguish *P. trisclerite* sp. nov. from *P. thayerae* by a number of morphological details of the head, pronotum, abdomen and legs (vide supra).

Although the abundance and diversity of Mesozoic staphylinids reflect the ancient origin and success of the family by the Late Cretaceous, e.g., [1,4,24,25,26,27,28,29,30,31], knowledge of osoriine palaeodiversity remains deficient [10,11]. In extant species of *Priochirus* the shapes of the cephalic horns (typically dubbed quite erroneously as “teeth”) are quite diverse, and it has been difficult to determine some aspects of their homology and variability [18,19]. Nonetheless, we noticed that the differences in cephalic characteristics between the species of *Eopriochirus* are not as great as differences between extant species, although the sample sizes are obviously trivial and therefore not much can be extrapolated from such an observation until further species and individuals are discovered. Wu and Zhou [18] suggested that the diversity of cephalic horns may result from different habitat specializations and strong selection, with osoriines perhaps evolving considerable disparity in their cephalic horns as a response to changes in habitat since the mid-Cretaceous. Forest litter may play an important role in the diversification of staphyliniform beetles [32], and the occurrence of osoriines amid forest litter and decomposing wood as these habitats evolved over the last 100 Ma may have resulted in shifts in cephalic architecture in relation to the use of the horns for pushing through the substrate and forest debris. Simultaneously, subcortical habitats likewise are associated with comparatively slow rates of change [25], perhaps accounting for the presence of an extant genus in the mid-Cretaceous amber of Myanmar. Cai et al. [33] suggested that the diversification rate and body size disparity among staphylinoid beetles through time was loosely correlated with changes in climate. The rapid radiation of staphylinoid beetles may be associated with occupying and diversifying refuge niches in low-energy conditions, rather than a response to the Cretaceous Terrestrial Revolution (KTR). At present, the available material of osoriines and several other critical subfamilies remain insufficient to critically evaluate these hypotheses, and we must await the discovery of further fossil material as well as an expanded knowledge of osoriine ecology and biology.

## Data Availability

No new data were created or analyzed in this study. Data sharing is not applicable to this article.
